# A_2A_R Antagonists Upregulate Expression of GS and GLAST in Rat Hypoxia Model

**DOI:** 10.1155/2020/2054293

**Published:** 2020-10-26

**Authors:** Jun Yu, Yan Yan, Yiye Chen, Yan Zheng, Xiaoyan Yu, Jialu Wang, Yafu Wang, Anken Wang, Xiaoli Kang, Jie Cen, Lingyan Dong, Li Li, Peiquan Zhao

**Affiliations:** ^1^Department of Ophthalmology, Xinhua Hospital, School of Medicine, Shanghai Jiao Tong University, Shanghai 200092, China; ^2^Department of Ophthalmology, Stanford University School of Medicine, Stanford, California 94303, USA; ^3^Department of Ophthalmology, Renji Hospital, School of Medicine, Shanghai Jiao Tong University, Shanghai 200127, China; ^4^Department of Ophthalmology, The Fifth People's Hospital of Shanghai, Shanghai 200240, China; ^5^Department of Ophthalmology, Shanghai Tenth People's Hospital, Affiliated Tongji University, Shanghai 200072, China

## Abstract

**Background:**

The aim of this study was to research the effects of glutamine synthetase (GS) and glutamate aspartate transporter (GLAST) in rat Müller cells and the effects of an adenosine A_2A_R antagonist (SCH 442416) on GS and GLAST in hypoxia both *in vivo* and *in vitro*.

**Methods:**

This study used RT-PCR and Western blotting to quantify the expressions of GS and GLAST under different hypoxic conditions as well as the expressions of GS and GLAST at different drug concentrations. A cell viability assay was used to assess drug toxicity.

**Results:**

mRNA and protein expression of GS and GLAST in hypoxia Group 24 h was significantly increased. mRNA and protein expressions of GS and GLAST both increased in Group 1 *μ*M SCH 442416 compared with other groups. One micromolar SCH 442416 could upregulate GS and GLAST's activity in hypoxia both *in vivo* and *in vitro*.

**Conclusions:**

Hypoxia activates GS and GLAST in rat retinal Müller cells in a short time *in vitro*. (2) A_2A_R antagonists upregulate the activity of GS and GLAST in hypoxia both *in vivo* and *in vitro*.

## 1. Introduction

The glymphatic system is a recently discovered waste clearance system in the brain that has been described as unique system of perivascular channels that are formed by astroglial cells. The glymphatic system promotes efficient elimination of soluble proteins and metabolites from the central nervous system (CNS) [1]. Additionally, the glymphatic system may help distribute non-waste compounds, such as glucose, amino acids, and neurotransmitters, that is related to volume transmission. As the chief astroglial cells in the retina, Müller cells play an important role. Müller cells are the predominant neuron-supporting glial cells in the retina; they span the entire retina and play an important role in the homeostasis of the retina by regulating the volume of the extracellular space, ion, water, and neurotransmitters, such as glutamate [2–4].

Glutamate is known to be a major excitatory neurotransmitter. However, under some pathological conditions (hypoxia, elevated intraocular pressure, ischemia, oedema, injury, etc.), increased glutamate accumulation will induce toxicity, which promotes death and degeneration of retinal ganglion cells (RGCS) [1, 4, 5]. Therefore, the balance of glutamate in the retina is crucial, and glutamate regulation depends on the glutamine cycle. Müller cells play a key role in this cycle. Excessive extracellular glutamate undergoes glutamate aspartate transporter- (GLAST-) mediated uptake into Müller cells and is detoxicated to glutamine quickly via glutamine synthetase (GS), a major enzyme in glutamate metabolism. Then, the atoxic glutamine is transported back to the external environment for glutamate resynthesis [4, 6].

Adenosine is a natural chemical messenger that is widely found in intracellular and extracellular fluids. Adenosine binds to G-protein-linked receptors which can be classified into the A_1_, A_2A_, A_2B_, and A_3_, 4 subtypes. It has been confirmed that all the four types of adenosine receptors exist in the retina [7–9]. Previous studies have demonstrated that A_2A_ receptor (A_2A_R) promotes the release of glutamate and that A_2A_R antagonists decrease its release. It has been suggested that A_2A_R antagonists might provide neuroprotection because they reduce the concentration of extracellular glutamate [9]. Our previous study also found that an A_2A_R antagonist (SCH 442416) upregulates GS and GLAST in Müller cells to improve the clearance of extracellular glutamate at elevated hydrostatic pressure *in vitro* [2].

In our present study, we investigate [1] the effect of GS and GLAST in rat retinal Müller cells in hypoxia and [2] the effect of an A_2A_R antagonist (SCH 442416) on GS and GLAST in hypoxia both *in vivo* and *in vitro*.

## 2. Materials and Methods

### 2.1. Cell Culture

The eyes of the Sprague-Dawley (new-born 0-3 days) rats were obtained from Shanghai Laboratory Animal Center CAS China (SLACCAS China). The method to prepare retinal tissue could is described our previous studies [2, 4, 7]. All retinal tissue was dissected and stored in phosphate buffer saline (PBS) (HyClone Beijing USA) on ice. The tissue was dissociated and incubated for 15 min in PBS containing 0.125% trypsin (Gibco USA) at 37°C and then added to Dulbecco's Modified Eagle Media: Nutrient Mixture *F-12* (DMEM/F12) medium (Gibco USA), which contained glutamine (2 mM), streptomycin (100 *μ*g/mL), and penicillin (100 U/mL). Ten percent foetal bovine serum (FBS) (HyClone USA) was used to terminate the digestion. The tissue was cultured in 10 mm dishes (in air containing 5% carbon dioxide, at 37°C) in an incubator (Thermo Germany). After the first outgrowth, Müller cells are one type of strong adherent cells. Other cell types (such as microglial cells and retinal ganglion cells) are poor adherents and could be rinsed off by exposure to PBS. Using this method, we obtained highly purified Müller cells. The medium was refreshed every 48 h.

Eight to ten days later, the cells were routinely cultured at 37°C and were transferred to a culture for a 2nd passage. Experiments were performed after the second passage when the confluence was 75–80%.

### 2.2. Hypoxia *In Vitro*

#### 2.2.1. Hypoxia Model

Hypoxia was initiated in a special chamber that had a controlled flow of a gas mixture (37°C, 94% nitrogen, 1% oxygen, and 5% carbon dioxide) **(**Thermo Germany).

### 2.3. Cultured Cell Treatment

#### 2.3.1. Searching for a Suitable Time-Point of GS and GLAST Activity

All of the cultures were added to six-well plates at 5 × 105/mL in serum-free DMEM (HyClone USA) for 6 h under normal condition (37°C, 20% oxygen, and 5% carbon dioxide). This method was used to homogenize the cells. Then, the hypoxia group cultures were placed in hypoxia model chambers for different times (12 h, 24 h, 36 h, and 48 h). The cultures of the normoxia groups were exposed to normal conditions (37°C, 20% oxygen, 5% carbon dioxide) for 48 h.

#### 2.3.2. Searching for a Suitable Drug Concentration for Animal Experiments

All of the cultures were added to six-well plates at 5 × 105/mL in serum-free DMEM (HyClone USA) for 6 h under normal conditions (37°C, 20% oxygen, 5% carbon dioxide). Then, the culture groups were placed in a hypoxia model chamber with different drug (SCH 442416) concentrations (0.1, 1, and 10 *μ*M) for a suitable amount of time to assess GS and GLAST activity.

### 2.4. Cell Viability Assay

The cells were cultured in 96-well culture plates. The density was 2 × 10^4^ cells/well. After 24 h, Cell Counting Kit-8 (CCK-8) (10 *μ*M/well) (Beyotime Shanghai China) was added to each well and incubated for 4 h. The absorbance at 450 nm was measured using a microplate reader. There were 8 groups (drug concentrations of control, 0.1, 1, and 10 *μ*M and normoxia or hypoxia).

### 2.5. Hypoxia *In Vivo*

#### 2.5.1. Hypoxia Model

**Figure 1:**
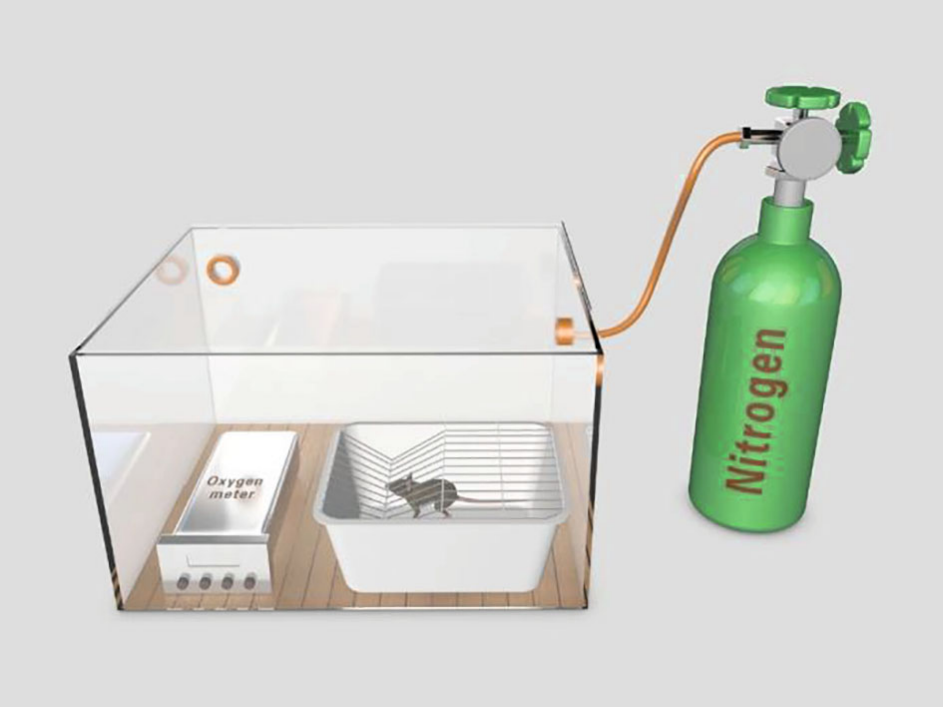
The hypoxia mechanism: It was a 60 × 80 cm plastic box. Nitrogen was pumped for adjusting the concentration of oxygen (10–12%) in the box. An oxygen meter was put for monitoring.

A special plastic box was designed. A 60 × 80 cm plastic box was used to construct the hypoxia mechanism (Figure 1). Nitrogen was pumped into the box to adjust the concentration of oxygen (10–12%). An oxygen meter was used to monitor the oxygen concentration.

#### 2.5.2. Intravitreal Injection

All of the described experiments comply with the National Institutes of Health Guidelines for Care and Use of Laboratory Animals. This study was approved by the animal ethics committee of Xinhua Hospital (Shanghai, China). Male Sprague-Dawley rats (200–250 g) were obtained from Shanghai Laboratory Animal Center CAS (Shanghai China) and were raised in a routine animal room. Rats were anesthetised with an intraperitoneal injection of xylazine (7.4 mg/mL) and ketamine hydrochloride (5 mg/kg) (Jiangsu Hergrui Medicine Co. Ltd) (Lianyungang China). The pupils of the rats were dilated with a tropicamide drop (Santen Japan). A 2 *μ*L solution (SCH 442416 or saline) was injected into the rat vitreous space (*n* = 4–6/group). Then, rats were seeded in a hypoxic box for 1 d, 3 d, and 5 d. Control group rats were intravitreally injected with the same amount of saline and were then seeded for 5 d.

### 2.6. Immunofluorescent Staining

#### 2.6.1. Cells

Expression of GS was determined with immunofluorescent staining. The cover slides were flushed first with PBS and fixed using sodium phosphate buffer (100 mM, pH 7.4), which contained 4% paraformaldehyde, for 10 min at 4°C. The cells were cultured with primary GS antibodies (Abcam British, 1 : 5000, polyclonal rabbit anti-GS antibody) overnight at 4°C. The cover slips were probed with fluorescein isothiocyanate Cy3- (BioLegend USA, 1 : 200) linked anti-rabbit IgG for 1 h at room temperature. The slides were cleared and photographed using an Axio microscope (Zeiss Germany).

#### 2.6.2. Rats

Rats were perfused with normal saline and a 4% paraformaldehyde (PFA) solution. The right eyes were covered with a 4% PFA solution for 4 h for fixation. Retinal tissue was vertically sectioned to thickness of 7.5 *μ*m, and then, 0.01 M PBS was added. The slices were blocked with 4% goat serum (Binyuntian China), 0.25% bovine serum albumin (HyClone USA), and 0.2% Triton X-100 (Binyuntian China) in PBS at room temperature for 2 h. The slices were incubated with a goat polyclonal anti-GLAST primary antibody (Santa Cruz USA, 1 : 200) and rabbit polyclonal anti-GS primary antibody (Santa Cruz USA, 1 : 400) at 4°C for 48 h. The retina slices were immunolabeled with fluorescein isothiocyanate (Invitrogen 1 : 200) or Cy3 (BioLegend USA, 1 : 200). Immunofluorescence images were generated with an imager laser-scanning microscope (Zeiss Germany).

### 2.7. Western Blotting Analysis

The method of handling retinal tissue has been described previously [2, 4, 7]. In brief, (1) extracted proteins extracted were boiled and centrifuged. (2) Proteins were separated and transferred to polyvinyl difluoride (PVDF) membranes (Millipore USA). (3) The membranes were soaked in Tris-buffered saline, which contained 5% skimmed milk and 0.1% Tween-20, for 1 h at room temperature. The primary antibodies used were for GS (Abcam British, 1 : 5000) and GLAST (Abcam British, 1 : 3000). The blots were incubated with primary antibodies overnight at 4°C. An anti-*β* actin antibody (Abcam British, 1 : 3000) was used as a reference to normalize the intensities of the immunoreactions with different antibodies. The membranes were incubated with a secondary antibody (Invitrogen USA, 1 : 10000) for 1 h at room temperature in darkness. The band intensities were quantified by scanning and densitometry with an Image Quant Las 4000 (GE USA).

### 2.8. Real-Time PCR Analysis

#### 2.8.1. RNA Extraction

The RNA extraction method has been described previously [2, 4, 7]. Total RNA from cultures was isolated by TRIzol reagent (Gibco USA) according to the manufacturer's instructions. RNA was treated with RNase-free DNase (Sangon Biotech China) to remove any genomic DNA contamination. The isolated RNA had an optical density (OD) 260/280 ratio of ≥2.0.

#### 2.8.2. Real-Time PCR

Two micrograms of total RNA was reverse-transcribed to a cDNA probe. The primer sequences were as follows: GS, sense 5′-ccgctcttcgtctcgttc-3′, antisense 5′-ctgcttgatgcctttgtt-3′; GLAST, sense 5′-cctatgtggcagtcgttt3′, antisense 5′-ctgtgatgggctggctaa3′; *β*-actin, sense 5′-cccatctatgagggttacgc-3′, antisense 5′-tttaatgtcacgcacgatttc-3. Real-time PCR was performed using a LightCycler instrument (ABI 7500 USA) with a SYBR-Green PCR Master mix (Takara Japan), according to the manufacturer's instructions.

### 2.9. Statistical Analysis

Data are presented as the mean ± standard error of the mean (SEM) (*n* = 4–6 each group). The data were analysed by ANOVA or Student's *t* test with the SPSS 18.0 software, and a *p* value < 0.05 was defined as significant.

## 3. Results

### 3.1. GS and Müller Cell

**Figure 2:**
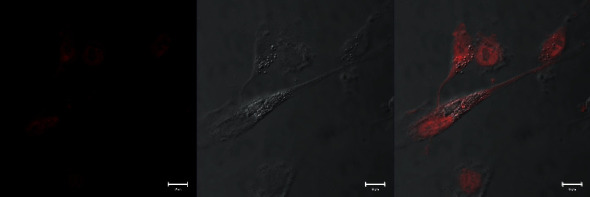
Identification of Müller cells. GS (red) was used to label Müller cells (×63).

GS is a specific molecular marker for Müller cells.  Figure 2 shows positive labelling for GS (Figure 2). GS is a specific molecular marker for Müller cells [1, 8–10]. In the present study, we found that >90% of culture cells were positive for GS, indicating that all of the cultured cells were Müller cells.

### 3.2. The Effect of Hypoxia on Expression of GS and GLAST

**Figure 3:**
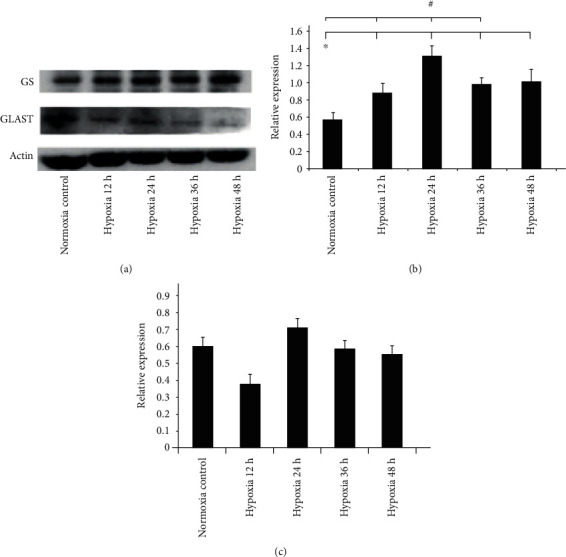
(a) GS and GLAST protein expression of Müller cells in different hypoxia groups. (b) GS mRNA expression of Müller cells in different hypoxia groups. (c) GLAST mRNA expression of Müller cells in different hypoxia groups.

From Western blotting analysis, we found that (1) protein expression of GS increased in hypoxia groups compared with the control group (normoxia), especially in the Group 24 h, and (2) although the protein expression of GLAST was weak, we also found that expression in Group 24 h was more obvious compared with that of other groups (Figure 3(a)).

PCR showed that GS mRNA fluctuated in the different hypoxia groups. All of the GS mRNA in the hypoxia groups (12 h, 24 h, 36 h, and 48 h) was increased compared with that of the control group (*p* = 0.043, *p* = 0.001, *p* = 0.004, and *p* = 0.034). The disparity of Group 24 h was the most obvious compared with other hypoxia groups (Figure 3(b)).

Unfortunately, we did not obtain a better result with GLAST mRNA (*p* = 0.491, *p* = 0.991, *p* = 1, and *p* = 1).  Figure 3(c) shows that GLAST mRNA decreased in Group 12 h; then, GLAST mRNA began to increase in Group 24 h. GLAST mRNA decreased later in Group 36 h and Group 48 h. Although the data were not significant, we found a trend indicating that hypoxia induced an increase in mRNA expression of GLAST in Group 24 h.

According to data analysis, we believed that hypoxic cell culture for 24 h was a suitable time-point for observing the activity of GS and GLAST.

### 3.3. The Effect of Concentration of SCH442416 on Expression of GS and GLAST in hypoxia

**Figure 4:**
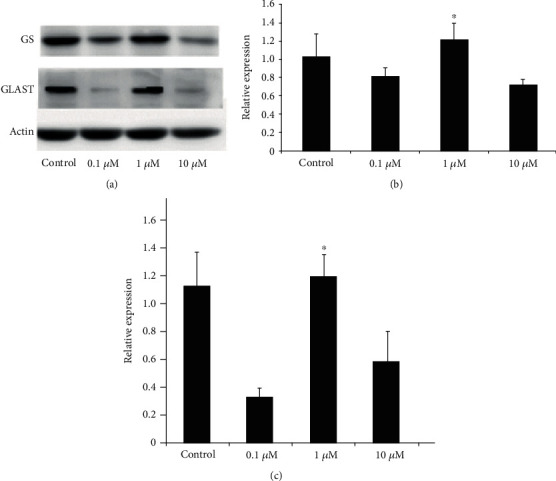
(a) GS and GLAST protein expression of Müller cells in different drug concentration groups. (b) GS mRNA expression of Müller cells in different drug concentration groups. (c) GLAST mRNA expression of Müller cells in different drug concentration groups.

From Western blotting analysis, we found that the GS and GLAST proteins both increased in Group 1 *μ*M (Figure 4(a)). PCR showed that GS and GLAST mRNA fluctuated at different drug concentrations. Group 1 *μ*M led to a higher mRNA concentration than the other groups (GS: *p* = 0.001, *p* = 0.001, and *p* = 0.001. GLAST: *p* = 0.001, *p* = 0.001, and *p* = 0.083) (Figures 4(b) and 4(c)).

**Figure 5:**
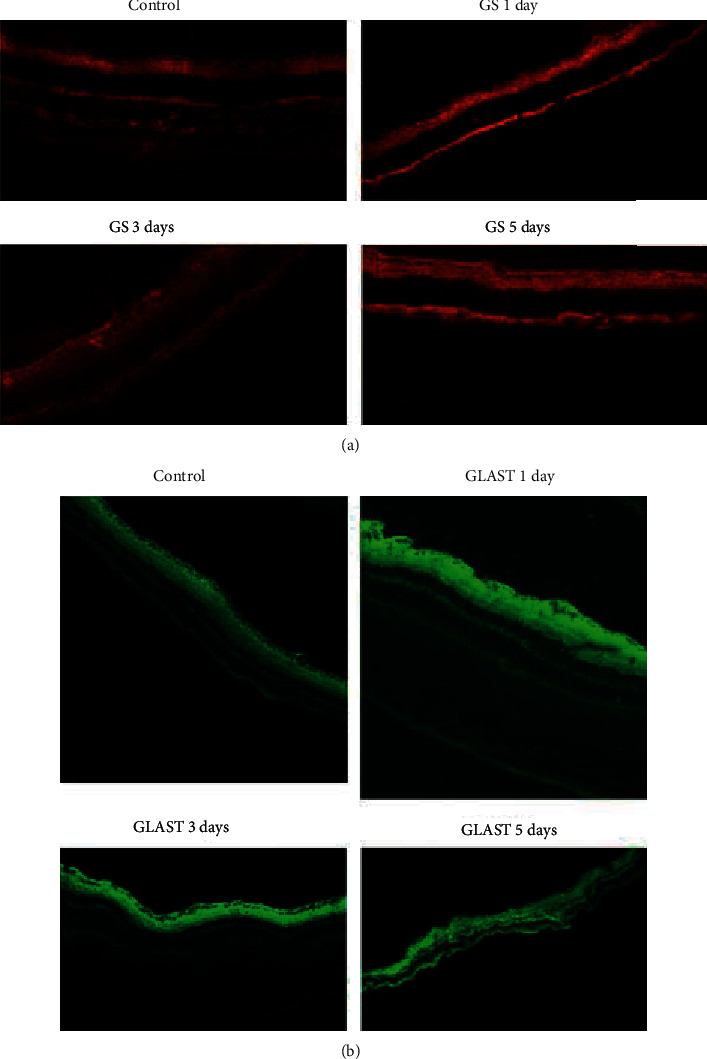
GS and GLAST protein expression.

A 1 *μ*M SCH 442416 solution was intravitreally injected into the right of rats. Rats were sacrificed at 1 d, 3 d, and 5 d. From the immunohistochemical experiments, compared with the control group that was injected with saline, GS and GLAST protein expression in group SCH 442416 in the retina increased over days (Figure 5).

### 3.4. The Drug Toxicity Test of SCH442416 for Müller Cells

The drug toxicity test showed that there was no significant difference in the SCH442416 groups (drug concentrations of 0.1, 1, and 10 *μ*M or normoxia or hypoxia) (normoxia: *p* = 0.536, *p* = 0.956, and *p* = 0.985. Hypoxia: *p* = 0.459, *p* = 0.082, and *p* = 0.263). Thus, the concentrations of SCH442416 (0.1, 1, and 10 *μ*M) used in our tests could not induce death of Müller cells.

**Figure 6:**
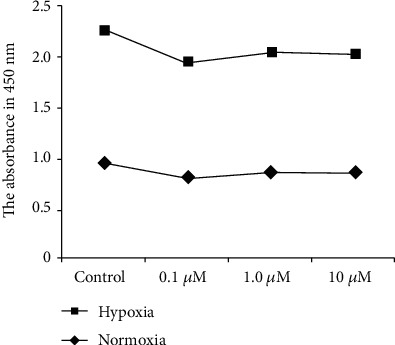
Müller cell viability assay test.

However, we found that the cell activity in all of the hypoxia groups was stronger than that in the normoxia groups. We inferred that hypoxia could enhance Müller cell activity (*p* = 0.001) (Figure 6).

## 4. Discussion

Our present results showed that hypoxia activated expression of GS and GLAST in rat retinal Müller cells. On base of the present and previous study, we found A_2A_R antagonists upregulated the expression of GS and GLAST in hypoxia both *in vivo* and *in vitro*.

Müller cells have be found accounted for 90% of the retinal gila [2–4]. And Müller cells span across the entire thickness of the retina. Müller cells constitute a link between the retinal neurons, the vitreous body, the retinal blood vessels, and the subretinal space anatomically and functionally. They have a wide array of responses to maintain homeostasis for neuronal and vascular elements. Just like a scavenger, they can maintain the integrity of the blood retinal barrier and clear metabolic waste in some pathological states (trauma, ischemia, high hydrostatic pressure, and hypoxia) [3, 4].

Retinal hypoxia plays a crucial role in a number of retinal diseases, such as diabetes, retinal oedema, retinal vascular disorders, and glaucoma [11]. Hypoxia is an important cause of CNS damage, which can result in excess excitatory amino acids, especially glutamate (a major neurotransmitter in the retina). Glutamate, which has a high concentration in retinal ganglion cells, leads to pathologic retinal ganglion cell death [11]. Some research found that hypoxia could induce upregulation of retinal glutamate uptake [12].

Glutamate is an important excitatory neurotransmitter and is also the primary mediator of excitatory synaptic transmission and excitotoxic neuronal injury in the retina. High concentrations of glutamate play a key role in retinal damage. Clearance of extracellular glutamate depends on the glutamate-glutamine cycle in Müller cells. GS and GLAST are the major enzymes in this cycle [13]. Thus, GS- and GLAST-mediated clearance of synaptic glutamate in Müller cells is critical because increased extracellular glutamate levels can lead to neurotoxicity through overstimulation of ionotropic glutamate receptors. GS and GLAST ultimately play an important role in regulating the balance between physiological signaling and pathological overactivation in the retina.

GS is a main enzyme in the metabolism of glutamate in glial cells. GS could catalyze the amidation of glutamate to glutamine, which is an important part of the circle of the transmitter pool of glutamate between neurons and glia. GS weak activity could lead to neuronal damage by allowing extracellular glutamate to accumulate. Decreased GS activity has also been reported after hypoxia or ischemia in the brain [4, 7]. GS can catalyze amidation of glutamate to glutamine in Müller cells. Krajnc et al. [14] found that GS mRNA remained elevated for over 6 h, but the activity of GS was low after more than 3 hours in the rat brain. Some studies reported that GS activity increased 24 h after hypoxia *in vitro* [15, 16]. In the present study, we found that hypoxia increased GS mRNA and protein in an earlier period *in vitro*. GS activity was highest at 24 h and later began to decrease.

Glutamate transporters are ultimately responsible for maintaining low extracellular glutamate concentration and thus play a crucial role in regulating the balance between physiological signaling and pathological overactivation. These five related glutamate transporters have been cloned: GLAST, GLT1 EAAC1, EAAT4, and EAAT5 [17]. GLAST is the main glutamate transporter for clearing excess glutamate from the extracellular milieu into Müller cells in the retina [17]. Previous research found that GLAST activity decreased in Müller cells in diabetic rat retina *in vitro* [18]. However, Mysona et al. [17] claimed that hyperglycaemia was insufficient to impair GLAST function in the short term. Some research indicated that GLAST was upregulated by hypoxia [19, 20], while other researchers presented the opposite results [21, 22]. Our present results suggested that although protein expression of GLAST was very weak, no significant difference was observed under different hypoxic conditions compared with GS. Hypoxia was also found to change the tendency of GLAST. Specifically, protein expression in group hypoxia 24 h was slightly higher than that of other hypoxia groups as was mRNA expression of GLAST. Thus, the suitable time-point of 24 h was determined for the A_2A_R antagonist (SCH 442416) experiments.

It was shown that all four subtypes of ARs (A_1_, A_2A_, A_2B_, and A_3_) were expressed in the retina. Blockade of A_2A_R affords robust neuroprotection in some noxious brain conditions, such as Huntington's disease, ischemia, and Alzheimer's disease [23, 24]. But there are no some reports about A_2A_R in retina. So we conducted a series of experiments. Our previous studies found that A_2A_R antagonists (SCH 442416) upregulate the expression of GLAST and GS in Müller cells and accelerate the clearance of extracellular glutamate at elevated hydrostatic pressure *in vitro* [2]. We observed similar outcomes in the present study, i.e., expression of GLAST and GS in Müller cells increased in hypoxia, not only *in vitro* but also *in vivo*.

In our animal hypoxia examination, we found that hypoxia induced the activity of GS and GLAST. Expression of GS and GLAST was further enhanced over days, indicating that A_2A_R antagonists increased the clearance of glutamate in the retina by strengthening the function of GS and GLAST. The time proceeded for 5 days after surgery. Although we studied changes in the retina during the early period, the time may have been too short for long-term observation.

## 5. Conclusions

In this study, hypoxia activates GS and GLAST in rat retinal Müller cells in a short period of time (12–48 hours) *in vitro*. Expression peaks at 24 hours. A_2A_R antagonists upregulate expression of GS and GLAST in hypoxia both *in vivo* and *in vitro*. And A_2A_R antagonists are safe for Müller cell. Hence, our results suggested A_2A_R antagonists might be a new drug for treatment of retinal diseases in the future.

## Data Availability

The data used to support the findings of this study are available from the corresponding author upon request.
